# A new *Diplolepis* Geoffroy (Hymenoptera, Cynipidae, Diplolepidini) species from China: a rare example of a rose gall-inducer of economic significance

**DOI:** 10.3897/zookeys.904.46547

**Published:** 2020-01-17

**Authors:** Juli Pujade-Villar, Yiping Wang, Wenli Zhang, Noel Mata-Casanova, Irene Lobato-Vila, Avar-Lehel Dénes, Zoltán László

**Affiliations:** 1 Department of Animal Biology, University of Barcelona, Barcelona 08028, Catalonia, Spain University of Barcelona Barcelona Spain; 2 College of Forest and Biotechnology, Zhejiang Agricultural and Forestry University, Lin’an 311300, China Zhejiang Agricultural and Forestry University Lin’an China; 3 Lanzhou Agro-technical research and Popularization Center, Lanzhou, 730010, China Lanzhou Agro-technical research and Popularization Center Lanzhou China; 4 Hungarian Department of Biology and Ecology, Babeș-Bolyai University, Cluj-Napoca 400006, Romania Babeș-Bolyai University Cluj-Napoca Romania

**Keywords:** gall wasp, Kushui rose, new species, phytophagous, taxonomy

## Abstract

A new species of the genus *Diplolepis* Geoffroy, *Diplolepis abei* Pujade-Villar & Wang **sp. nov.** is described on host plant *Rosa sertata* Rolfe × *R. rugosa* Thunb. from China with an integrative approach based on molecular and morphological data. Diagnosis, distribution and biology of the new species are included and illustrated. This species is the first known rose gall-inducer of economic importance. A review of Eastern Palearctic species of *Diplolepis* is given and a key to the Chinese fauna is presented.

## Introduction

The family Cynipidae (Hymenoptera, Cynipoidea) includes around 1400 species, all of them exclusively phytophagous and divided into 12 tribes (Ronquist 1999; Ronquist et al. 2015). The tribe Diplolepidini induces galls exclusively on *Rosa* spp. L. and is distributed in the Holarctic Region. Diplolepidini is a monophyletic group characterized by a unique autapomorphy within Cynipidae: the presence of a longitudinal furrow on the mesopleuron of the mesosoma. Only two genera, *Diplolepis* Geoffroy, 1762 and *Liebelia* Kieffer, 1903, are currently included in this tribe, together comprising 58–62 species (Melika 2006; Ronquist et al. 2015). *Diplolepis* and *Liebelia* can be easily distinguished on the basis of the following diagnostic morphological characters (Melika 2006): in *Diplolepis*, antennae of females and males are 12–15-segmented, the radial cell of the forewing is closed along the wing margin and the hypopygium is plough-shaped, while in *Liebelia* antennae are 16-segmented, the radial cell is open and the hypopygium is not plough-shaped.

The genus *Diplolepis* includes six European species (Pujade-Villar 1993; Pujade-Villar and Plantard 2002), some of them also occurring in Western Asia and North Africa (Morocco) (Mimeur 1949; Melika 2006); eight Eastern Palaearctic species (Abe et al. 2007; Wang et al. 2013) and 31 North American species (Burks 1979; Shorthouse and Ritchie 1984). The relatively low number of species recorded from the Eastern Palaearctic could be due to a lack of sampling efforts; herein we describe a new species for the Eastern Palaearctic fauna.

Guo et al. (2013) and Wang et al. (2013) published the first record of *D. rosae* in China, which later has been proved to be a misidentification. The eastern boundary of *D. rosae*’s distribution range may be the Ural, Altay, Tianshan, Pamir, Hindukush Mountains (Belizin 1957). However, a single mention of a more eastern location: India, appears in the key of Belizin (1957: 11), a record that has not been confirmed since then. Therefore, we presume that *D. rosae* does not occur in China.

*Diplolepis* is morphologically characterized by having both the head and the mesosoma black or reddish brown; antennae 14–15 segmented (except *D. hunanensis* with only 12) in both sexes and with relatively large, cylindrical flagellomeres; pronotum dorsomedially short; pronotal plate not pronounced; scutellar foveae faint or absent; mesopleuron with a broad, crenulate mesopleural furrow; propodeum rugose and lateral propodeal carinae usually indistinct; metanotal trough broad, apically truncate; forewings moderately or strongly uniformly or partially infuscate, margins with short but distinct cilia; radial cell closed along the wing margin; 2r of forewings usually with a prominent median vein stump anterolaterally projected; nucha dorsally short; hypopygium plough-shaped and hypopygial spine slightly longer than broad, with some sparse short setae (Melika 2006; Ronquist et al. 2015).

*Diplolepis* are known to cause galls mainly on feral roses. Accidental infections on cultivated *Rosa rugosa* and its hybrids were reported from North America, but neither of the *Diplolepis* species became serious pests of rose cultivars (Shorthouse 2001). There are no published data on the large-scale economic importance of *Diplolepis*.

## Material and methods

The terminology for the morphology of cynipid gall wasps used in this work follows Liljeblad and Ronquist (1998) and Melika (2006). Abbreviations for the forewing venation are taken from Ronquist and Nordlander (1989), and the cuticular surface terminology, from Harris (1979). Measurements and abbreviations used in this work are: F1–F12, first and subsequent flagellomeres; post-ocellar distance (POL), the distance between the inner margins of the posterior ocelli; ocellar-ocular distance (OOL), the distance from the outer margin of the posterior ocellus to the inner margin of the compound eye; LOL, the distance between lateral and frontal ocelli. The width of the forewing radial cell was measured from the margin of the wing to the Rs vein.

Scanning electron microscope (SEM) images of the described species were taken with a FEI Quanta 200 ESEM at high voltage (15 kV) without gold coating in the “Serveis de Microscopia Electrònica” of the University of Barcelona.

Specimens of the new species were collected in Lanzhou City of Gansu Province and they are deposited in the Hymenoptera Collection of Zhejiang Agricultural and Forest University (**ZAFU**) and in the University of Barcelona (UB, Col. JP-V).

Genomic DNA was extracted from two individuals using an ISOLATE II Genomic DNA Kit (Bioline, Germany), following the protocol provided by the manufacturer. The mitochondrial cytochrome c oxidase subunit I (COI) sequences were amplified using the standard LCO1490 and HCO2198 primer pair (Folmer et al. 1994) in a 50 µl reaction volume at a 42 °C annealing temperature. PCR products were purified with the Wizard SV Gel and PCR Clean-Up System (Promega, USA) and sent for sequencing to Macrogen Inc. (Europe).

Sequences were downloaded and verified with the BLAST (Johnson et al. 2008). Further, sequences for all available *Diplolepis* species were also downloaded from the NCBI database and the BOLD System (see Fig. 5 for reference numbers). The sequences were aligned using a Clustal W algorithm (Thompson et al. 1994) in BioEdit (Hall 1999). A Bayesian inference (BI) tree was generated in MrBayes (Ronquist et al. 2012) for 1,000,000 generations, sampled every 1000^th^ step, until the average standard deviation of split frequencies fell below 0.01. The first 25% of the sampled trees were discarded as burn-in. The GTR+G+I model of molecular evolution was selected as most suitable for this analysis, based on the Bayesian information criterion in jModelTest2 (Darriba et al. 2012). Interspecific *p*-distances were calculated in MEGA X (Kumar et al. 2018).

## Results

### Diplolepis abei

Taxon classificationAnimaliaGentianalesApocynaceae

Pujade-Villar & Wang sp. nov.

CED897BB-ACA9-57F5-B8FE-3167B3BB07F4

http://zoobank.org/E35C7053-8145-4CAC-B453-C6A657A11330

Figs 1 , 2

#### Type material.

***Holotype***: ♀ deposited in UB with the following labels: ‘Lanzhou (Gansu Province), ex *Rosa sertata* × *R. rugosa*, (03.ii.2011) 15.iii.2011, col. Sheng Maoling’ (white label); ‘*Diplolepis abei* Pujade-Villar & Wang, desig. JP-V-2017’ (red label). ***Paratypes***: 11♀♀ with the same labels as the holotype: 8♀♀ in ZAFU, 3♀♀ in UB.

#### Diagnosis.

This species is characterized by having the following morphological characters: head smooth to alutaceous, mesoscutum alutaceous with piliferous punctures, scutellum rugose with a more delicate sculpture in the centre of the disk; legs, including coxae, reddish; forewings hyaline but slightly smoky in both the radial and the 3^rd^ cubital cells, never with a dusky cloud around veins; second metasomal tergite short. It differs from the rest of species known from China because veins of its forewings are not infuscate. In addition, the deciduous galls have numerous long stout sharp-pointed spines unlike other known species. Molecular results: the two sequenced individuals represent one haplotype (GeneBank accession number: MN434062). Based on the BI tree the species is part of a polytomous clade with a group consisting of *D. fructuum*, *D. mayri* and *D. rosae*, and with *D. spinosissimae* (Fig. 5). The average *p*-distance compared to the other species is 9.73% (Table 1), with the lowest values shown when compared to *D. fructuum* (6.38%) and *D. spinosissimae* (6.39%).

**Table 1.** P-distance values for sequences of *Diplolepis abei* sp. nov. and all available *Diplolepis* species. Abbreviations: *dradi*: *Diplolepis radicum*, *drosfol*: *D. rosaefolii*, *dfusif*: *D. fusiformans*, *dtrif*: *D. triforma*, *dspinsa*: *D. spinosa*, *degla*: *D. eglanteriae*, *dbic*: *D. bicolor*, *dbass*: *D. bassetti*, *doreg*: *D. oregonensis*, *dignot*: *D. ignota*, *dvari*: *D. variabilis*, *dgrac*: *D. gracilis*, *dpoli*: *D. polita*, *dnodu*: *D. nodulosa*, *dnebu*: *D. nebulosa*, *dfruc*: *D. fructuum*, *dcalif*: *D. californica*, *dmayr*: *D. mayri*, *drosa*: *D. rosae*, *dnerv*: *D. nervosa*, *dspinsi*: *D. spinosissimae*, *dspnov*: *D. abei* sp. nov.

	***dradi***	***drosfol***	***dfusif***	***dtrif***	***dspinsa***	***degla***	***dbic***	***dbass***	***doreg***	***dignot***	***dvari***	***dgrac***	***dpoli***	***dnodu***	***dnebu***	***dfruc***	***dcalif***	***dmayr***	***drosa***	***dnerv***	***dspinsi***
*dradi*																					
*drosfol*	13.14																				
*dfusif*	11.81	4.55																			
*dtrif*	6.54	11.98	10.87																		
*dspinsa*	6.60	12.59	11.87	7.15																	
*degla*	15.25	13.90	12.81	13.81	14.31																
*dbic*	14.17	11.54	9.57	13.56	14.73	12.06															
*dbass*	14.59	11.36	9.48	13.14	14.98	12.31	6.24														
*doreg*	6.99	12.63	10.62	7.82	8.90	13.56	11.81	12.48													
*dignot*	11.59	10.93	9.32	10.48	11.98	11.98	10.40	11.81	10.15												
*dvari*	11.92	10.75	9.15	10.48	11.98	11.98	10.40	11.81	10.15	0.83											
*dgrac*	13.09	9.97	8.65	11.48	12.31	12.65	10.40	10.65	10.98	5.66	5.49										
*dpoli*	14.14	11.13	8.71	12.65	13.70	12.42	5.68	2.61	11.56	10.59	10.59	9.93									
*dnodu*	7.04	11.68	10.76	5.71	7.60	15.09	13.84	13.87	8.21	10.87	10.87	11.20	12.92								
*dnebu*	11.76	10.75	9.15	10.32	12.15	11.81	10.23	11.65	9.98	0.17	0.67	5.49	10.43	10.70							
*dfruc*	11.76	9.62	8.32	10.82	12.48	12.81	9.40	9.15	9.48	8.65	8.65	9.48	7.82	10.21	8.49						
*dcalif*	5.75	12.38	11.13	6.58	6.66	14.24	14.32	14.32	7.70	11.49	11.74	11.49	13.43	6.86	11.66	10.99					
*dmayr*	11.00	8.60	7.54	10.39	12.23	12.06	9.13	8.88	9.38	8.71	8.71	9.38	7.76	10.44	8.54	2.35	10.39				
*drosa*	11.33	9.21	8.21	10.80	12.81	12.40	9.72	9.72	10.18	8.71	8.71	9.55	8.71	10.78	8.54	3.02	10.90	2.09			
*dnerv*	13.46	10.27	9.76	12.39	13.69	10.23	10.83	11.44	13.78	11.09	11.09	12.48	10.80	13.06	10.92	10.23	12.40	9.71	10.14		
*dspinsi*	12.27	9.36	8.67	11.48	13.52	12.50	8.84	8.50	12.25	7.91	8.08	9.61	7.71	10.85	7.91	6.03	11.91	5.61	6.29	9.62	
*dspn*	12.89	10.13	8.89	12.00	13.03	12.26	9.15	10.10	11.05	8.81	8.63	8.80	8.49	11.54	8.63	6.38	12.19	6.90	7.94	10.11	6.39

**Description. Female. *Length*.** Body length 3.3–3.6 mm (*N* = 4).

***Color*** (Fig. 2g). Head and mesosoma uniformly black. Antenna black; pedicel, and sometimes also the scapus, lighter. Tegulae brown. Mandibles reddish, with black tips, maxillary and labial palpi brown. Legs reddish, including coxae; last tarsi (and sometimes the 4^th^ tarsomere) darker. Metasoma reddish; basal and posterior parts and hypopygium, brown. Wings hyaline but slightly smoky in both the radial and the 3^rd^ cubital cells; wing veins distinct, dark brown, never with infuscate clouds.

**Figure 1.**
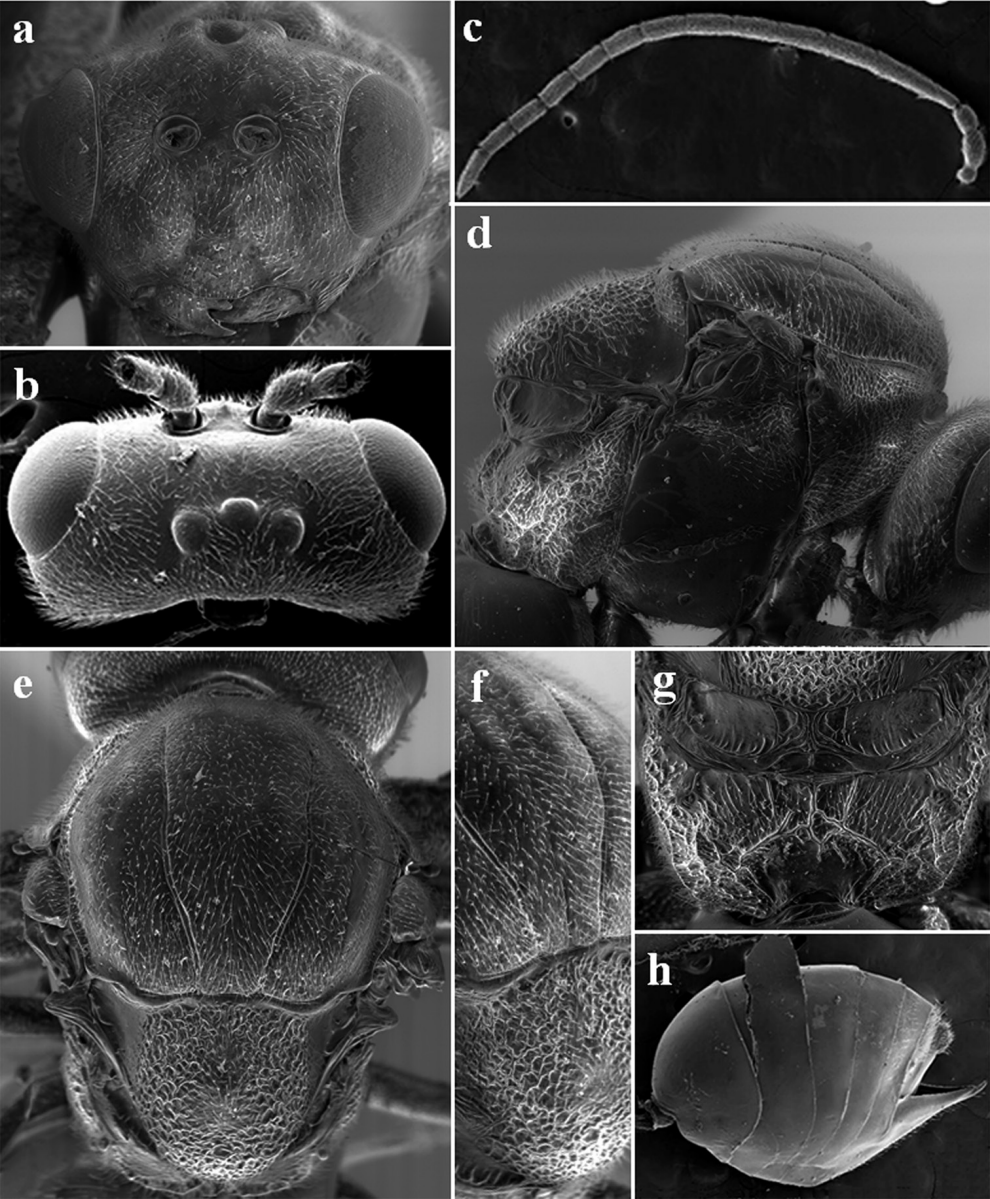
*Diplolepis abei* Pujade-Villar & Wang ♀ sp. nov. **a** head in frontal view **b** genae in dorsal view **c** antenna **d** mesosoma in lateral view **e** mesosoma in dorsal view **f** mesosoma in dorso-lateral view **g** propodeum **h** metasoma in lateral view.

***Head*** (Fig. 1a, b). Head trapezoidal in frontal view, transverse, as wide as the mesosoma, shiny, with short sparse white setae, 1.3 times as broad as high in frontal view and 2.1 times as broad as long seen from above. Lower face smooth to alutaceous, with distinct piliferous punctures; median elevated area alutaceous. Clypeus quadrangular, broader than high, smooth to alutaceous, flattened; anterior tentorial pits, epistomal sulcus and clypeo-pleurostomal line, distinct, ventral margin straight. Gena smooth to alutaceous, with piliferous punctures and basally with some weak carinae, not broadened behind the compound eye (not visible in frontal view) and 2.0 times as broad as the cross diameter of the compound eye in lateral view. Malar space smooth to coriaceous, around 0.5 times as long as height of compound eye. Transfacial distance 1.5 times as long as height of compound eye; diameter of antennal toruli 1.4 times as long as the distance between them, and distance between torulus and eye margin 1.2 times longer than torulus diameter. Inner margins of compound eyes divergent. Frons and vertex shiny, alutaceous; occiput dull, coriaceous. POL 0.75 times as long as OOL; OOL 2.0 times longer than the diameter of the lateral ocelli and 6.6 times longer than LOL.

**Figure 2.**
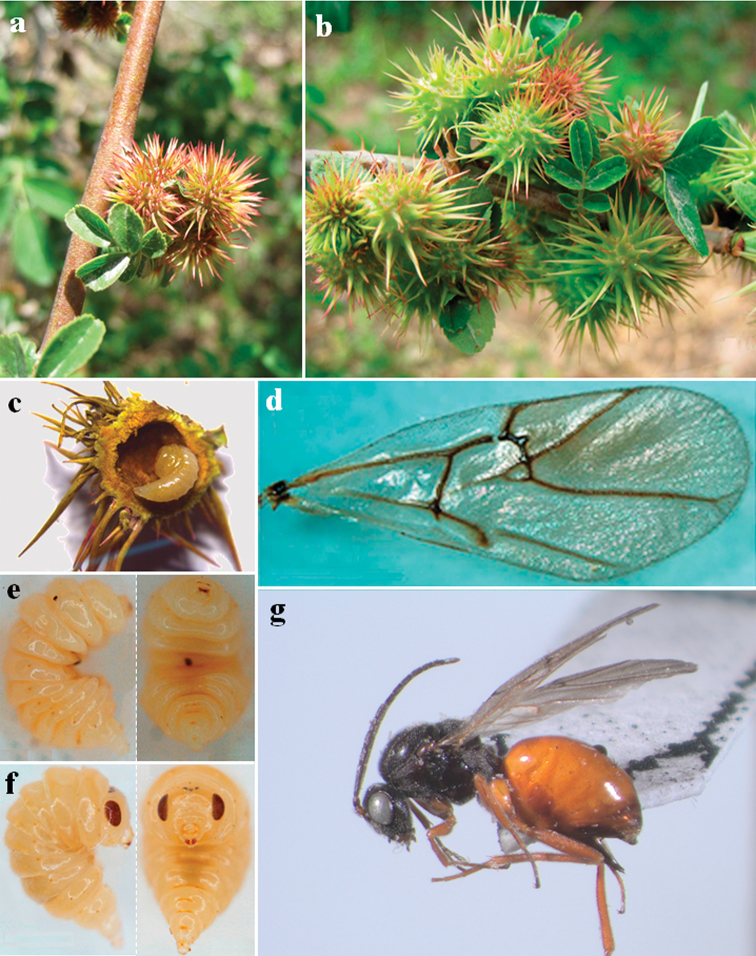
*Diplolepis abei* Pujade-Villar & Wang ♀ sp. nov. **a–b** galls **c** dissected gall with last instar larva **d** forewing **e** lateral and ventral view of the 3^rd^ instar larva **f** lateral and ventral view of the last instar larva **g** lateral habitus.

***Antenna*** (Fig. 1c). 14-segmented, 1.5 times longer than head plus mesosoma; pedicel slightly longer than broad; F1 very long, 4.0 times longer than pedicel and nearly 1.8 times longer than F2; F2 as long as F3; F12 slightly longer than F11; placodeal sensilla present in all the funicular segments, but only apically in F1 and in the anterior half in F2. Antennal formula: 6: 4(x3): 16: 9: 9: 8: 8: 8: 7: 7: 7: 7: 7: 9.

***Mesosoma*** (Fig. 1d–g). Mesosoma curved and slightly longer than high in lateral view, with short white setae. Pronotum very narrow, coarsely punctured, sparsely haired in the middle and coriaceous with some carinae in the basal part. Scutum wider than long and at least 1.7 times longer than the scutellum, alutaceous, with distinct punctures. Notauli complete, convergent posteriorly; median mesoscutal line very shallow, reaching at least the level of tegulae; parapsidal lines visible but poorly impressed, narrow, shining, reaching tegulae level; anterior parallel lines distinct, smooth, extending to half the length of the scutum. Scutellum as long as wide, with parallel lateral margins, rounded posteriorly, dull, rugose, with a more delicate sculpture in the centre of the disk. Scutellar foveae short, transversal, inconspicuous, rugose, not delimited posteriorly. Mesopleuron smooth and shiny, with a strong transverse dull rugose furrow; mesopleural triangle with numerous delicate wrinkles. Metapleural sulcus reaching the mesopleuron slightly above half of its height; axillula ovate, smooth, distally with some short rugae, without setae; subaxillular bar coriaceous, with very delicate carinae. Dorsellum smooth with some carinae, inferiorly convex. Metanotal trough smooth, shining, with some longitudinal parallel weak wrinkles and without setae; ventral impressed area alutaceous, without delicate longitudinal wrinkles, shining. Propodeum laterally rugose, medially smooth; lateral propodeal carinae anteriorly with three straight carinae and strongly curved outwards in posterior 2/3, delimiting a closed area.

***Legs*.** Tarsal claws simple, without a basal lobe.

***Forewing*** (Fig. 2d). Radial cell partially closed and margin pigmented, 2.3 times longer than wide, first abscissa of radius nearly straight, 2r with an additional median prolongation into the radial cell. Areolet distinct, large. Rs+M well-marked and reaching basalis in the lower third.

***Metasoma*** (Fig. 1h). Slightly longer than head plus mesosoma length (1.1×); in lateral view, 1.4 times longer than high. Second metasomal tergite short, reaching 1/3 if the metasoma; metasomal tergites 2 to 5 without punctures, subsequent tergites alutaceous. Hypopygium plough-shaped, shiny, smooth and large; prominent part of the ventral spine of the hypopygium very thin, 3.0 times longer than broad, with sparse white setae, apical setae short, not extending behind apex of the spine.

**Male**: unknown.

***Gall*** (Fig. 2a–c). Resembles the North American gall *Diplolepis bicolor* (Harris, 1841), but the new species has more abundant spines and a different coloration. It also resembles *D. japonica* (Walker, 1874), but the shape and the length of the spines are very different. The galls of the new species are spherical-shaped, appearing as monothalamous or one-celled swellings bearing numerous long, stout and sharp-pointed spines that are longer than the diameter of the galls. Their surface is smooth and glabrous. Galls arise on branches, buds or leaf veins of *Rosa sertata* Rolfe × *R. rugosa* Thunb., usually in groups. Young galls are pea green or reddish green and soft, gradually turning greyish green and harder when maturing. The inner cell is large, and the delimiting wall of parenchymatous cells is thick, usually 1.5 mm thick. Mature galls are deciduous.

#### Host.

The new species was collected on the Chinese Kushui rose, a hybrid of *Rosa sertata* Rolfe × *R. rugosa* Thunb. which is cultivated mainly in Gansu Province (China) for its oil. *Rosa rugosa* also occurs at the collection site (a Kushui rose plantation) but no galls were found on them despite growing only a few meters from Kushui roses supporting large numbers of galls. To the best of our knowledge this may be the first known *Diplolepis* species that causes significant agricultural loss. In Gansu Province (China) the *R. sertata* × *R. rugosa* hybrid is commonly planted for its high yields of flowers and oil. The infected shrubs may suffer up to 70% yield loss according to rose oil farmers (We et al. 2014). In infected plantations, *D. abei* is considered a significant pest which reduces rose flower numbers and subsequent rose oil yields.

#### Biology.

Only females are known (Fig. 2g). Galls appear in mid-April and larvae occupy the most part of the larval chamber (Fig. 2c, e, f). Adults emerge in early March of the following year.

#### Comment.

In Wang et al. (2013) and Guo et al. (2013), the material corresponding to this new species is determined as *D. rosae*. In Wang et al. (2013) seven males and nine females were cited. The reason why there are more females (12) in the present paper than those mentioned in Wang et al. (2013) is that the sexes were confused in Wang et al. (2013): four of the specimens were considered males, although they were females. The other four specimens of the 16 mentioned in Wang et al. (2013) are lost.

#### Distribution.

China (Gansu Province).

#### Etymology.

Named in honour of the Japanese cynipidologist and friend, Prof. Yoshihisa Abe (Biosystematics Laboratory, Graduate School of Social and Cultural Studies, Kyushu University, Fukuoka, Japan).

## Discussion

**Figure 3.**
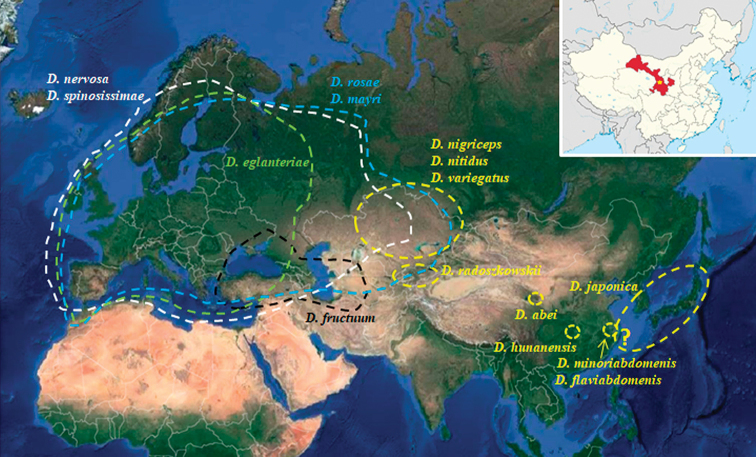
Distribution of the 11 species of *Diplolepis* of the Palearctic; in yellow, the species distributed exclusively in the Eastern Palearctic. Palaearctic map obtained from https://www.google.com/maps/@57.7164944,49.0396796,9792440m/data=!3m1!1e3. The inset image pointing out in red the Gansu Province (and thus the collecting location) was obtained from https://en.wikipedia.org/wiki/Gansu.

According to Abe et al. (2007), *D. brunneipes* (Ashmead, 1904) from Japan has an uncertain status, and *D. kunugi* Shinji, 1938, also from Japan, is not a *Diplolepis*. Thus, based on Abe et al. (2007) a total of 11 species occur in the Palaearctic Region of which five are distributed in the Eastern Palearctic (Fig. 3). Wang et al. (2013) subsequently described three new *Diplolepis* species from China, increasing the number of recorded species present in the Eastern Palaearctic to eight: *D. japonica* (Walker, 1874) from Japan and possibly also from China (see comments below); *D. nigriceps* Vyrzhikovskaja, 1963, *D. nitidus* Vyrzhikovskaja, 1963 and *D. variegatus* Vyrzhikovskaja, 1963 from Kazakhstan; *D. radoszkowskii* Kieffer, 1904 from Uzbekistan and Tajikistan; and *D. flaviabdomenis* Wang, Liw & Chen, 2013, *D. hunanensis* Wang, Liw & Chen, 2013 and *D. minoriabdomenis* Wang, Liw & Chen, 2013 from China). From Asia Minor and Western Palearctic region six species are known: *D. fructuum* (Rübsaamen, 1895) from Turkmenistan; *D. mayri* (Schlechtendal, 1876) from Siberia, Kazakhstan, Turkmenistan, Uzbekistan, Tajikistan and Kyrgyzstan; *D. nervosa* (Curtis, 1838) (= *D. centifoliae* (Hartig, 1840)) from Western Kazakhstan; *D. rosae* (Linnaeus, 1758) from Southern Kazakhstan, Turkmenistan, Uzbekistan, Tajikistan and Kyrgyzstan; *D. spinosissimae* (Giraud, 1859) from Kazakhstan; and a single species, *D. eglanteriae* (Hartig, 1840), from Europe and North Africa (Morocco).

*Diplolepis rosae* is also recorded from India (Belizin 1957), which must be confirmed, and erroneously from China (Guo et al. 2013; Wang et al. 2013) – this record was a misidentification of the new species described here. Records of *D. nervosa* and *D. spinosissimae* by Kovalev (1965) must be confirmed too. The only *D. japonica* specimen mentioned from China (Wang et al. 2013) collected in Malaise trap presents some differences in the sculpture with respect the redescription of *D. japonica* provided by Yasumatsu and Taketani (1967) (see taxonomic key below); for this reason, we consider this specimen here as Diplolepis nr japonica, pending collection of more specimens and its gall. Diplolepis nr japonica could be an undescribed species.

The new species, *D. abei* Pujade-Villar & Wang induces spherical galls with spines resembling *D. japonica* and, some forms of *D. nervosa* in the Palaearctic Region. However, *D. abei* differs from these species by producing galls with relatively longer, pointed, hard and woody spines. Adults of *D. nervosa* differ from the new species by having POL slightly longer than OOL, the scutum coriaceous, the scutellum strongly elongated (nearly 2.0 times longer than wide) with subparallel margins and slightly constricted basally, the scutellar foveae present (large, transversely ovate and smooth) and forewings are hyaline (neither with infuscate areas nor smoky marks); on the other hand, in *D. japonica* the radial cell is shorter (around 2.0 times as long as wide), forewings are hardly infuscate around radial cell, the face is coarsely rugose, the mesoscutum is smooth and the 2^nd^ tergite occupies more than half the length of metasoma.

**Figure 4.**
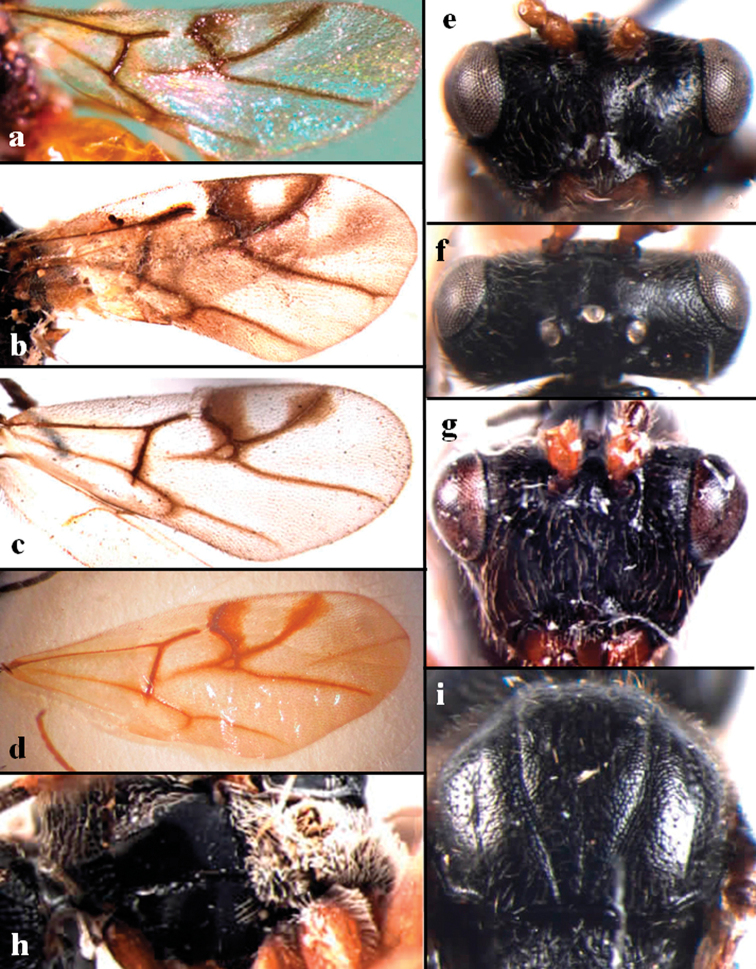
**a** forewing of *D. flaviabdomenis***b** forewing of *D. hunanensis***c** forewing of *D. minoriabdomenis*, and **d** forewing of D. nr japonica**e** head in frontal view of D. nr japonica (reused from Wang et al. 2013) **f** head in dorsal view of D. nr japonica (reused from Wang et al. 2013) **g** head in frontal view of *D. hunanensis***h** lateral mesosoma of *D. minoriabdomenis***i** mesoscutum of D. nr japonica.

**Figure 5.**
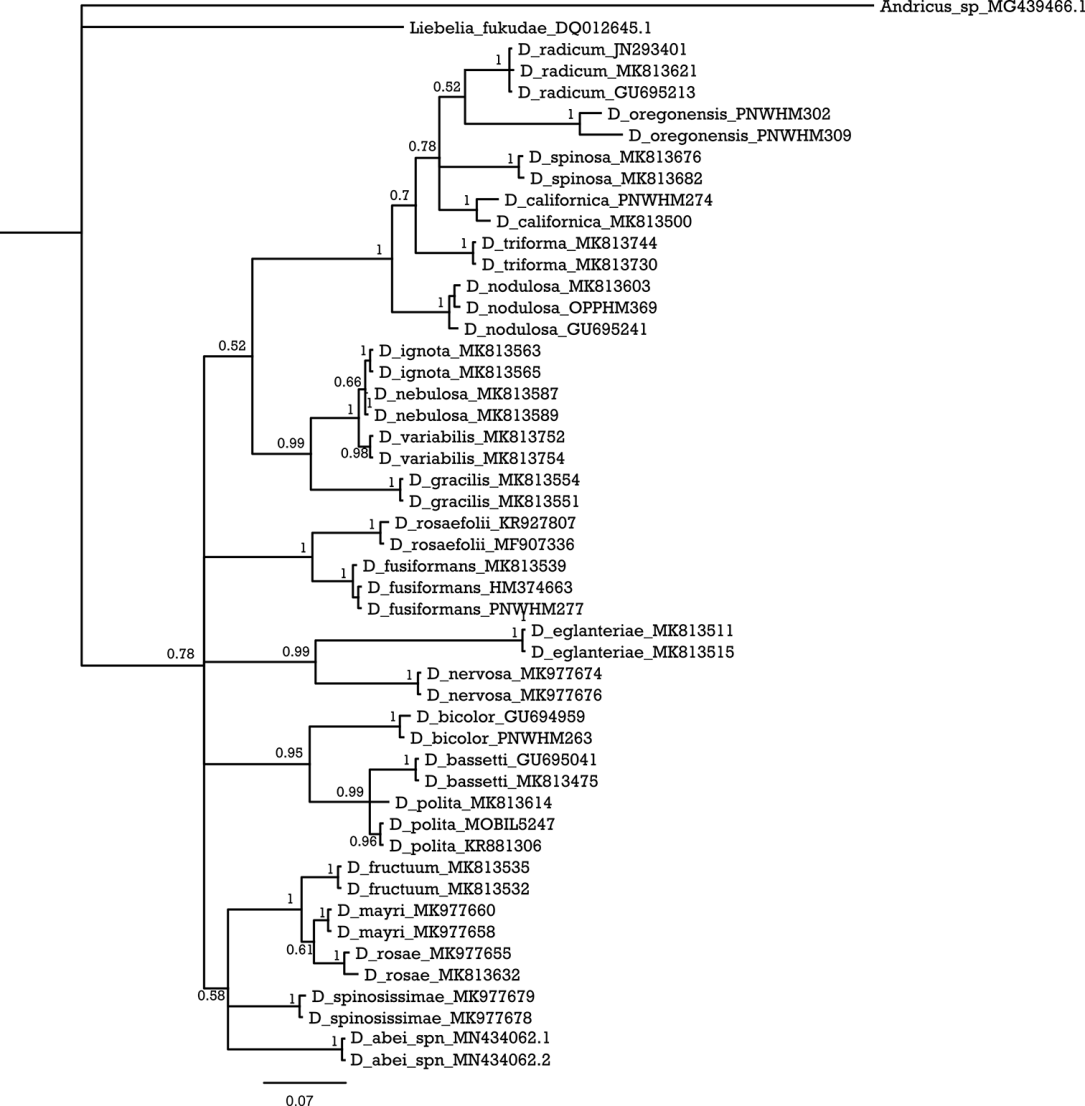
Bayesian inference (BI) tree of the *Diplolepis* species that have available mitochondrial COI sequences. Numbers on the branches represent posterior probabilities (PP).

### The species of *Diplolepis* present in China can be differentiated from each other according to the following key:

**Table:** 

1	Radial cell relatively long, longer than 2.5 times as long as broad (Figs 2d, 4a)	**2**
–	Radial cell shorter, around 2.0 times as long as broad (Fig. 4b–d)	**3**
2	Radial cell closed, with infuscate veins and 2r vein without projection into the radial cell (Fig. 4a). Malar distance long, around 0.75 times as long as compound eye height	***D. flaviabdomenis***
–	Radial cell partially open in margin, without infuscate veins and 2r vein with a projection into the radial cell (Fig. 2d). Malar distance shorter, around 0.5 times as long as compound eye height	***D. abei* sp. nov.**
3	Head strongly transverse in frontal view, 1.7 times wider than high (Fig. 4e) and slightly wider than mesosoma. Median mesoscutal line faintly present posteriorly over 1/4 of the entire length of mesoscutum. Occiput sculptured but never with striae. Propodeum sparsely setose	**4**
–	Head trapezoid-shaped in frontal view, around 1.5 times wider than high (Fig. 4g) and distinctly narrower than mesosoma. Median mesoscutal line absent or only present by a very short depression (extending over 1/10 of mesoscutum length). Occiput smooth and shiny with striae. Propodeum densely pubescent (Fig. 4h)	**5**
4	Vertex and mesoscutum smooth and shiny. Occiput coarsely punctured. From Japan and Korea	***D. japonica***
–	Vertex (Fig. 4f) and mesoscutum (Fig. 4i) distinctly alutaceous to coriaceous. Occiput coriaceous. From China	***D. japonica***
5	Antennae 12-segmented, with scapus and pedicel yellowish-brown. POL around 2.0 times longer than OOL. Parapsidal lines absent, almost invisible. Radial cell closed (Fig. 4b). Third and following metasomal tergites with distinct punctures dorso-laterally	***D. hunanensis***
–	Antennae 14-segmented, with scapus and pedicel black. POL around 3.0 times longer than OOL. Parapsidal lines distinct and extending almost the entirely length of mesoscutum. Radial cell completely open in margin (Fig. 4c). All metasomal tergites without punctures	***D. minoriabdomenis***

*Diplolepis abei* is the first *Diplolepis* associated with a gall from China; *D. flaviabdomenis*, *D. hunanensis* and *D. minoriabdomenis* were described from material collected by Malaise traps (Wang et al. 2013). This new species occurs on *R. sertata × rugosa*, and there are around 100 described species of *Rosa* in China (Wu et al. 2003) of which at least 65 species are endemic (eFloras 2008); therefore, the richness of Diplolepidini (*Diplolepis* and *Liebelia*) is probably greater. As an example of how poorly understood *Diplolepis* is in Eastern Palaearctic, a species of *Periclistus* Förster, 1869, which are obligate inquilines of *Diplolepis*, was recently described from China (Pujade-Villar et al. 2015). Its host remains unidentified, but the gall morphology differs from that of *D. abei*.

Finally, *D. abei* Pujade-Villar & Wang is morphologically closely related to ‘*rosae*’ clade according to Pujade-Villar and Plantard (2002). This clade includes four Western Palaearctic species: *D. rosae*, *D. mayri*, *D. fructuum* and *D. spinosissimae*. It is defined morphologically, according to Pujade-Villar (1993), by the following characters: scutellum rounded, medial sulcus absent or rudimentary (in species with smoky wing areas) or present (in species without strongly smoky areas), F1 at least 1.7 times F2, straight in females (curved and shortly expanded in males), head in frontal view oval and galls not detachable from plant tissues. The ‘*rosae*’ clade has been also confirmed by Zhang et al. (2019) as the ‘Palaearctic multi-chamber subclade’. The closeness of *D. abei* to the ‘*rosae*’ clade is also confirmed by the molecular genetic results based on COI sequences. It is the first species of this group present in China.

## Supplementary Material

XML Treatment for Diplolepis abei
